# Energy Expenditure Estimation During Crutch-Orthosis-Assisted Gait of a Spinal-Cord-Injured Subject

**DOI:** 10.3389/fnbot.2019.00055

**Published:** 2019-07-18

**Authors:** Florian Michaud, Francisco Mouzo, Urbano Lugrís, Javier Cuadrado

**Affiliations:** Laboratory of Mechanical Engineering, University of La Coruña, Ferrol, Spain

**Keywords:** energy expenditure, SCI subject, crutch-assisted gait, KAFO, human modeling and analysis, muscle recruitment problem

## Abstract

Determination of muscle energy expenditure by computer modeling and analysis is of great interest to estimate the whole body energy consumption, while avoiding the complex character of *in vivo* experimental measurements for some subjects or activities. In previous papers, the authors presented optimization methods for estimating muscle forces in spinal-cord-injured (SCI) subjects performing crutch-assisted gait. Starting from those results, this work addresses the estimation of the whole body energy consumption of a SCI subject during crutch-assisted gait using the models of human muscle energy expenditure proposed by Umberger and Bhargava. First, the two methods were applied to the gait of a healthy subject, and experimentally validated by means of a portable gas analyzer in several 5-min tests. Then, both methods were used for a SCI subject during crutch-assisted gait wearing either a passive or an active knee-ankle foot orthosis (KAFO), in order to compare the energetic efficiency of both gait-assistive devices. Improved gait pattern and reduced energy consumption were the results of using the actuated gait device. Computer modeling and analysis can provide valuable indicators, as energy consumption, to assess the impact of assistive devices in patients without the need for long and uncomfortable experimental tests.

## Introduction

In the last decade, many mechanical and, more recently, electromechanical (or hybrid) devices have been developed to allow spinal-cord-injured (SCI) patients to stand and walk (White et al., 2014; Cuadrado et al., 2019). At the moment, the additional use of crutches is generally required for gait stability. Despite these technological advances, most SCI subjects prefer the wheelchair to move for energetic efficiency reasons (Merati et al., 2000). The gait efficiency can be defined as the percentage of energy input that is transformed into useful work. Use of a cane or a pair of crutches requires about 33% more energy than normal walking (Mcbeath et al., 1974). In addition, some devices (KAFO), don't allow some joints to move, which implies another gait pattern even less efficient. Moreover, since structures of the upper extremities are designed primarily for prehensile activities, not to walk, many patients suffer from shoulder and arm injuries (Lee and McMahon, 2002).

Energy cost in subjects using crutches was mainly studied by means of experimental measurements (Mcbeath et al., 1974; Waters and Mulroy, 1999; Merati et al., 2000), generally using a gas analyzer. IJzerman et al. (1998) proposed an alternative method to estimate the energy expenditure of paraplegic gait using measurements of heart rate and crutch forces. In all the previous methods, the patient must go through experimental tests lasting several minutes while wearing not only the assistive devices, but also measuring devices as the gas analyzer, which is rather uncomfortable. This may be too demanding for many patients. Conversely, the method proposed in this paper just requires the motion-force-EMG capture of a gait cycle, which is much more achievable for most patients. Using the captured data, a musculoskeletal model of the subject provides the joint efforts and muscle forces, activations and excitations and, then, models of human muscle energy expenditure proposed in the literature are applied to the results to estimate the energy cost of the measured gait.

Various Hill-based models can be found in the literature to calculate the human muscle energy expenditure (Minetti and Alexander, 1997; Umberger et al., 2003; Bhargava et al., 2004; Houdijk et al., 2006). Miller proposed a comparison of these models for the gait of healthy subjects (Miller, 2014). According to his recommendations, the models of Umberger and Bhargava have been implemented in this work to calculate the energy cost of SCI subjects during crutch gait. Since both muscle energy expenditure models are based on the Hill's muscle model, they require the knowledge of some muscular parameters. Such parameters had been obtained by the authors in a previous work (Michaud et al., 2017), using physiological static optimization (Ou, 2012), and a customized musculoskeletal model of the SCI subject.

The objective of this work is to estimate the energetic cost of the crutch-orthosis-assisted gait of a SCI subject so that comparison from the energetic point of view may be established between two assistive devices: a passive and an active KAFO. The latter was obtained by simply adding to the passive device a motor and gearbox at knee level and an inertial sensor at shank level, so that motion intention is detected, and knee flexion/extension is automatically produced during swing. First, the methods for energetic cost estimation were applied to the gait of a healthy subject, and experimentally validated by means of a portable gas analyzer on several 5-min tests. Then the same methods were applied to the SCI subject.

The motivation of the work comes from the fact that walking is essential for the general health state of SCI subjects, thus overcoming the sedentarism due to permanent use of the wheelchair. Orthotic devices enable some SCI subjects to walk, but sometimes the energetic cost of the resulting gait is so high that patients reject this option. Therefore, evaluation of the energetic cost of gait allows to assess, even since the early training period, whether a certain orthotic device is promising for actual use by the patient in the mid and long terms. Moreover, it can provide valuable data to track the training progress. However, experimental estimation of energetic cost through 5-min tests is not feasible in most cases and, then, the alternative of getting an acceptable estimation from a short motion/force/EMG capture appears as greatly interesting.

Contributions of the paper are: (i) the detailed description of Umberger's and Bhargava's methods for the estimation of energetic cost, providing all the necessary elements required for implementation of the methods; (ii) the experimental validation of both methods for healthy gait by comparison with the results obtained from 5-min tests; (iii) the application of both methods to crutch-orthosis-assisted gait for the cases of passive and active orthoses; (iv) the comparison, in terms of energetic cost, between assisted gait with passive and active orthoses.

The remaining of the paper is organized as follows: section Materials and Methods describes the experiments and models used in this work; section Results presents the two energy expenditure models implemented; and sections Discussion and Conclusion provide, respectively, the obtained results and their corresponding discussion.

## Materials and Methods

### Subjects

The SCI subject was a 49-years-old male of mass 82 kg and height 1.90 m, with injury corresponding to a Lower Extremity Muscle Score (LEMS) of 13/50. His injury allowed him a normal motion of the upper extremities and trunk, while partially limiting the actuation at the hips and right knee due to partial or no muscular innervation. Both motor and sensory functions at ankles and left knee were totally lost. Therefore, in order to walk he required the assistance of a passive KAFO at the left leg, a passive ankle-foot orthosis at the right leg and two forearm crutches. However, permanent left-knee extension, even during the swing phase, made gait become very uncomfortable as hip raising was required for swing, thus demanding high efforts which led to fatigue quickly. Consequently, in daily life he mainly used a wheelchair to move and resorted to the mentioned assisted gait only occasionally and during short periods of time.

To improve SCI subjects' mobility, a research prototype of a low-cost active KAFO was developed (Font-Llagunes et al., 2016). Starting from a conventional passive device, an electric motor (EC90 flat of 160 W) plus a Harmonic gearbox (CCD-P-20-100-C-I with a ratio of 100:1) was placed at knee level substituting the external original joint, so as to launch the swing cycle when motion intention was detected by an inertial sensor placed at shank level, in order to avoid foot-collision with the ground. After some training sessions, the subject was able to walk with the mentioned prototype, featuring an actuated left-knee flexion of 30 degrees.

In order to assess the subject's muscle activity at hip and knee levels, surface EMG measurements were taken during simple exercises.

The healthy subject was a 28-years-old male of mass 85 kg and height 1.87 m.

### Instrumentation and Data Collection

**Figure 1:**
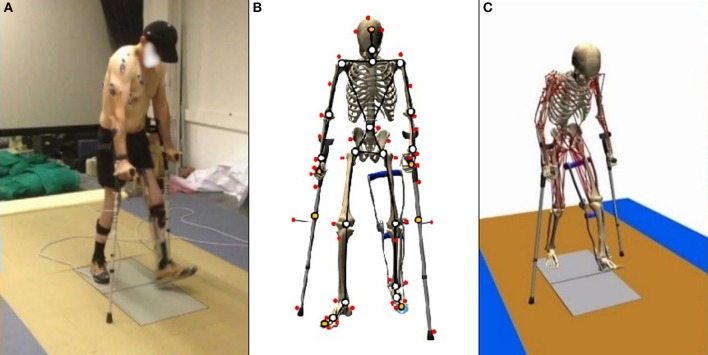
Gait of SCI subject assisted by passive orthoses and crutches: **(A)** motion-force-EMG capture; **(B)** skeletal model; and **(C)** musculoskeletal model.

**Figure 2:**
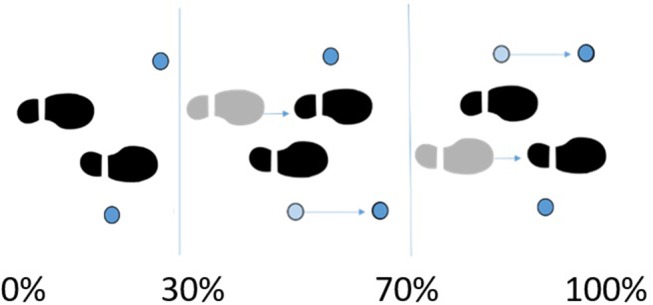
2-point crutch-assisted gait cycle.

**Figure 3:**
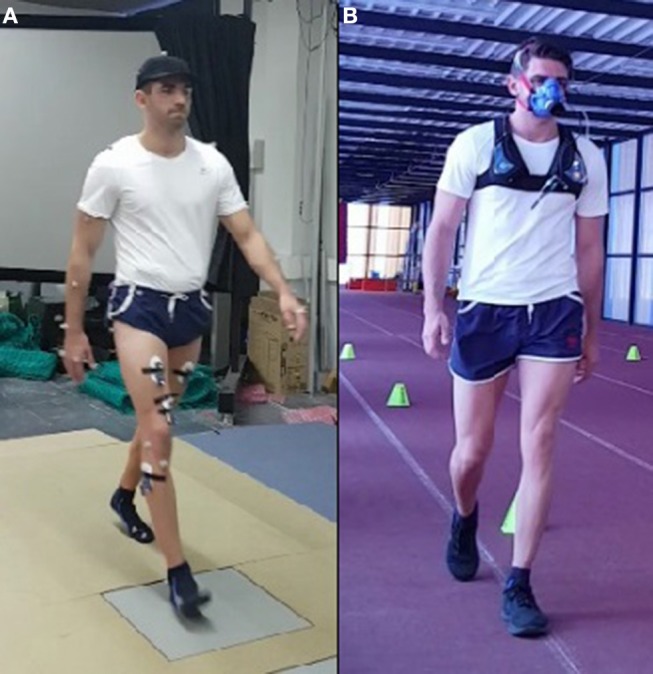
Energy consumption for a healthy subject: **(A)** motion-force-EMG capture; **(B)** 5-min test with portable gas analyzer. (Written informed consent was obtained from the individual for the publication of these images. FlM, the main author, is on this picture).

Subjects walked over two embedded force plates (AMTI, AccuGait, sampling at 100 Hz), with the help of two instrumented crutches for ground reaction measurement in the case of the SCI subject (Lugris et al., 2013), while their motion was captured by 12 optical infrared cameras that computed the position of 37 optical markers attached to the subjects' body, and 3 more for each crutch in the case of the SCI subject. Moreover, 10 EMG signals were recorded (2 at the right leg, 3 at the trunk, 4 at the right arm, and 1 at the left arm) for the SCI subject and 10 for the healthy subject at the lower extremities (Figures 1A, 3A). A complete gait cycle was captured of the SCI subject walking with (i) the passive orthosis owned by the subject; (ii) the active orthosis with the motor locking the knee; (iii) the active orthosis with the motor moving the knee. He used the 2-point crutch-assisted gait cycle shown in Figure 2.

For the healthy subject, 21 complete gait cycles were recorded at seven different speeds (between the free selected speed and fast speed) for energetic cost calculation. The energy expenditure was also measured experimentally by means of a portable gas analyzer (Cortex MetaMax 3B) during two 5-min tests at free selected speed and fast speed (Figure 3B). This experimental method requires that the subject maintains a constant speed during at least 5 min. Since this was thought to be too demanding for SCI subjects, it was decided to carry out the experimental validation with a healthy subject.

Calculations were performed on an Intel® Core™ i7–6,700 K, at 4.00 GHz with 16 Gb of RAM.

### Model Description

For the healthy subject, the human 3D model consisted of 18 anatomical segments: pelvis, torso, neck, head, and two hind feet, forefeet, shanks, thighs, arms, forearms and hands. For the SCI subject (Figure 1B), the same model was used, but the hands were rigidly connected to the crutches, and the orthosis at the left leg was embedded in the corresponding body links (thigh, calve, and foot). The segments were linked by ideal spherical joints, thus defining a model with 57° of freedom (6 of the base body plus 17 × 3 of the joints). The geometric and inertial parameters of the model were obtained, for the lower limbs, by applying correlation equations from a reduced set of measurements taken on the subject, following the procedures described in Vaughan et al. (1999). For the upper part of the body, data from standard tables (Ambrosio and Kecskemethy, 2007) was scaled according to the mass, and height of the subject. In order to adjust the total mass of the subject, a second scaling was applied to the inertial parameters of the upper part of the body. Assistive devices were taken into account by altering the inertia properties of hands (crutches) and thigh, calve and foot (orthosis). Mixed (natural and joint) coordinates along with matrix-R formulation (de Jalon and Bayo, 1994) were applied to obtain the joint drive torques along the motion using the in-house developed MBSLIM library (Dopico, 2016) programmed in FORTRAN language.

**Figure 4:**
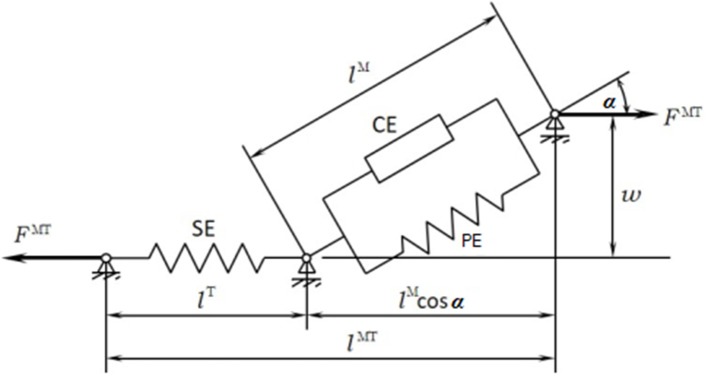
Hill's muscle model.

The musculoskeletal model was customized to the SCI subject according to his muscle activity (previously measured through EMG). The musculoskeletal model (Figure 1C) was composed of 112 muscles for the whole body: 28 at the right hip, 5 at the right knee, 21 at the left hip, 6 at the trunk, 15 at each shoulder, and 11 at each elbow. For the healthy subject, only the lower extremities were considered with their 92 muscles (43 muscles per leg plus 6 at trunk); the energy consumption of upper body muscles was considered into the basal energy consumption. Muscle properties were taken from Delp et al. (2007). The Hill's muscle model shown in Figure 4 was employed, being considered both the tendon and the muscle, with its contractile (CE), and passive (PE) elements. The muscle recruitment problem was addressed by means of the physiological static optimization method (Ou, 2012) using in-house developed code programmed in Matlab, and calling to *fmincon* Matlab's function for optimization, thus getting the histories of muscle forces, activations and excitations. Energy expenditure calculations were also programmed in the same in-house code.

### Energy Expenditure

Once muscular activity obtained as previously explained, results were validated with the experimental EMG measurements. The obtained activation, length, velocity and force of the muscles were used as input for the two models of energy expenditure. Both of them are based on the first law of thermodynamics. According to this law, the total rate of energy consumption $\dot{E}$, is equal to the rate at which heat is liberated, $\dot{H}$, plus the rate at which work is done, $\dot{W}$:

(1) $$\begin{array}{r} \dot{E}=\dot{H}+\dot{W} \end{array}$$

#### Umberger's Model

Umberger's muscle energy expenditure model (Umberger et al., 2003) considers the activation heat rate (${\dot{h}}_{A}$), the maintenance heat rate (${\dot{h}}_{M}$), the shortening/lengthening heat rate (${\dot{h}}_{SL}$), and the mechanical work rate of the contractile element of the muscle (${\dot{w}}_{CE}$), to determine the total rate of muscle energy expenditure ($\dot{E}$). The relation is given by the sum of this four terms expressed in (2), where $\dot{E}$ is calculated for each muscle in W.kg^−1^.

(2) $$\begin{array}{r} \dot{E}={\dot{h}}_{A}+{\dot{h}}_{M}+{\dot{h}}_{SL}+{\dot{w}}_{CE} \end{array}$$

##### Activation and maintenance heat rate

A combined expression of the activation and maintenance heat rate is used for this first term,

(3) $$\begin{array}{r} {\dot{h}}_{A}+{\dot{h}}_{M}={\dot{h}}_{AM}=1.28\times \%FT+25 \end{array}$$

where *%FT* represents the percentage of fast twitch found in Johnson et al. (1973).

##### Shortening and lengthening heat rate

During CE shortening (***V***_***M***_**(*t*)** ≤ 0) and lengthening (***V***_***M***_(*t*) > 0), the rate of heat production is modeled as the product of a coefficient **α**_***S***_ and ***V***_***M***_, the velocity of the muscular contractile element:

(4) $${\dot{h}}_{SL}(t)=\{\begin{array}{l} -\alpha_{S(ST)}{\tilde{V}}_{M}(t)(1-\%FT/100)-\alpha_{S(FT)}{\tilde{V}}_{M}(t)(\%FT/100)\text{ if }V_{M}(t)\leq 0\\ \alpha_{L}{\tilde{V}}_{M}(t)\text{ if }V_{M}(t)>0 \end{array}$$

with the constant terms $\alpha_{S(ST)}=\frac{4\times 25}{{\tilde{V}}_{M(MAX-ST)}},$ $\alpha_{S(FT)}=\frac{153}{{\tilde{V}}_{M(MAX-FT)}},$ $\alpha_{L}=4\alpha_{S(ST)},$ ${\tilde{V}}_{M}=\frac{V_{M}}{l_{0}^{M}},$ ${\tilde{V}}_{M(MAX-FT)}=\frac{V_{MAX}^{M}}{l_{0}^{M}},$ ${\tilde{V}}_{M(MAX-ST)}={\tilde{V}}_{M(MAX-FT)}/2.5,$ and $V_{MAX}^{M}=l_{0}^{M}/0.1$ ($l_{0}^{M}$ the optimal fiber length).

##### Mechanical work rate

The specific mechanical work rate is given by:

(5) $$\begin{array}{r} {\dot{w}}_{CE}\left(t\right)=-\left(F_{CE}^{M}\left(t\right)V_{M}\left(t\right)\right)/m \end{array}$$

so that this value is positive for concentric effort and negative for eccentric effort. ***m*** represents the mass of the muscle.

##### Total energy expenditure scaled

Equation (2) provides the energy expenditure of the muscle for the case of full activation and the contractile element length of the muscle (***l***^***M***^) is equal to the optimal muscular length ($l_{0}^{M}$) of the contractile element. Scaling factors are needed to account for the length and activation dependence of ${\dot{h}}_{AM}$ (dependence factor ***A***_***AM***_) and ${\dot{h}}_{SL}$ (dependence factor ***A***_***SL***_), and the dependence of the total heat rate on the metabolic working conditions (*S* = 1 for primarily anaerobic conditions and *S* = 1.5 for primarily aerobic conditions),

(6) $$\dot{E}(t)=\{\begin{array}{l} {\dot{h}}_{AM}A_{AM}(t)S+{\dot{h}}_{SL}(t)A_{SL}(t)S+{\dot{w}}_{CE}(t)\text{ if }l^{M}(t)\leq l_{0}^{M}\\ (0.4\times {\dot{h}}_{AM}+0.6\times {\dot{h}}_{AM}\times F_{0}^{M})A_{AM}(t)S+{\dot{h}}_{SL}(t)A_{SL}(t)S+{\dot{w}}_{CE}(t)\text{ if }l^{M}(t)>l_{0}^{M} \end{array}$$

with $A_{AM}\left(t\right)=A{\left(t\right)}^{0.6}$, $A_{SL}\left(t\right)=A{\left(t\right)}^{2}$, and

(7) $$A(t)=\{\begin{array}{ll} u(t) & \text{if }u(t)\leq a(t)\\ (u(t)+a(t))/2 & \text{if }u(t)>a(t) \end{array}$$

where ***u***(***t***) and ***a***(***t***) represent the excitation and activation of the muscle, respectively.

#### Bhargava's Model

Bhargava's model presents some similarities with the previous one, since the general expression is similar to equation (2) with an additional a basal metabolic rate ${\dot{h}}_{B}$:

(8) $$\begin{array}{r} \dot{E}={\dot{h}}_{A}+{\dot{h}}_{M}+{\dot{h}}_{SL}+{\dot{w}}_{CE}+{\dot{h}}_{B} \end{array}$$

However, expressions of the components are slightly different.

##### Activation heat rate

(9) $$\begin{array}{r} {\dot{h}}_{A}=\phi f_{FT}{\dot{A}}_{FT}u_{FT}(t)+\phi f_{ST}{\dot{A}}_{ST}u_{ST}(t) \end{array}$$

with

(10) $$\begin{array}{r} \phi =0.06+\exp(-t_{stim}u(t)/\tau_{\phi }), \end{array}$$

(11) $$\begin{array}{r} u_{FT}(t)=1-\cos(\frac{\pi }{2}u(t)) \end{array}$$

and

(12) $$\begin{array}{r} u_{ST}(t)=\sin(\frac{\pi }{2}u(t)), \end{array}$$

and the constant terms: ***f***_***FT***_ = %***FT***/100, ***f***_***ST***_ = 1 − %***FT***/100, ${\dot{A}}_{FT}$ = 133 and ${\dot{A}}_{ST}$ = 40.

##### Maintenance heat rate

(13) $$\begin{array}{r} {\dot{h}}_{M}\left(t\right)=L\left({\tilde{l}}^{M}\left(t\right)\right)f_{FT}{\dot{M}}_{FT}u_{FT}\left(t\right)+L\left({\tilde{l}}^{M}\left(t\right)\right)f_{ST}{\dot{M}}_{ST}u_{ST}\left(t\right) \end{array}$$

where $L\left({\tilde{l}}^{M}\left(t\right)\right)$ is a function that models the dependence on muscle length:

(14) $$L({\tilde{l}}^{M}(t))=\{\begin{array}{ll} 0.5 & \text{if }{\tilde{l}}^{M}(t)\leq 0.5\\ \;{\tilde{l}}^{M}(t) & \text{if }0.5<{\tilde{l}}^{M}(t)\leq 1\\ -2({\tilde{l}}^{M}(t))+3\; & \text{if }1<{\tilde{l}}^{M}(t)\leq 1.5\\ 0 & \text{if }{\tilde{l}}^{M}(t)>1.5 \end{array}$$

with ${\tilde{l}}^{M}=l^{M}/l_{0}^{M}$ and the maintenance heat rate constants: ${\dot{M}}_{FT}=111$ and ${\dot{M}}_{ST}=74$.

##### Shortening and lengthening heat rate

During CE shortening and lengthening, the rate of heat production is modeled as the product of a coefficient **α**_***S***_ and ***V***_***M***_, as it happened in Umberger's model,

(15) $$\begin{array}{r} {\dot{h}}_{SL}(t)=-\alpha_{S}(t){\tilde{V}}_{M}(t). \end{array}$$

However, expression of **α**_***S***_ is different:

(16) $$\alpha_{S}(t)=\{\begin{array}{ll} 0.16F_{0}^{M}+0.18F_{CE}^{M}(t) & \text{if }V_{M}(t)\leq 0\\ 0.157F_{CE}^{M}(t) & \text{if }V_{M}(t)>0 \end{array}$$

##### Basal heat rate

In addition, Bhargava's model proposes a basal metabolic rate calculated from a frog skeletal model at 0°C and given by:

(17) $$\begin{array}{r} {\dot{h}}_{B}=0.0225 \end{array}$$

##### Mechanical work rate

Both models consider the same expression for the mechanical work rate:

(18) $$\begin{array}{r} {\dot{w}}_{CE}\left(t\right)=-\left(F_{CE}^{M}\left(t\right)V_{M}\left(t\right)\right)/m \end{array}$$

#### Total Energy Consumption

Finally, the total energy consumption $\dot{E}$ of the full body during a full stride was obtained for both models by:

(19) $$\begin{array}{r} \dot{E}=\left(\overset{n}{\underset{i=1}{\sum }}\left(\frac{\overset{t_{cycle}}{\underset{t=0}{\int }}\left({\dot{E}}_{i}\left(t\right)\times m_{i}\right)dt}{t_{cycle}}\right)+k_{B}\times m_{residual}\right)/m_{subject} \end{array}$$

where ***m***_***subject***_ is to the mass of the subject, ***n*** the number of muscles, ***t***_***cycle***_ the time of a gait cycle, and

(20) $$\begin{array}{r} m_{residual}=m_{subject}-\overset{n}{\underset{i=1}{\sum }}m_{\;i} \end{array}$$

Lastly, ***k***_***B***_ represents the basal added metabolic rate of 1.2 W.kg^−1^ which corresponds to the energy consumption for upright quiet standing (Waters and Mulroy, 1999).

## Results

**Figure 5:**
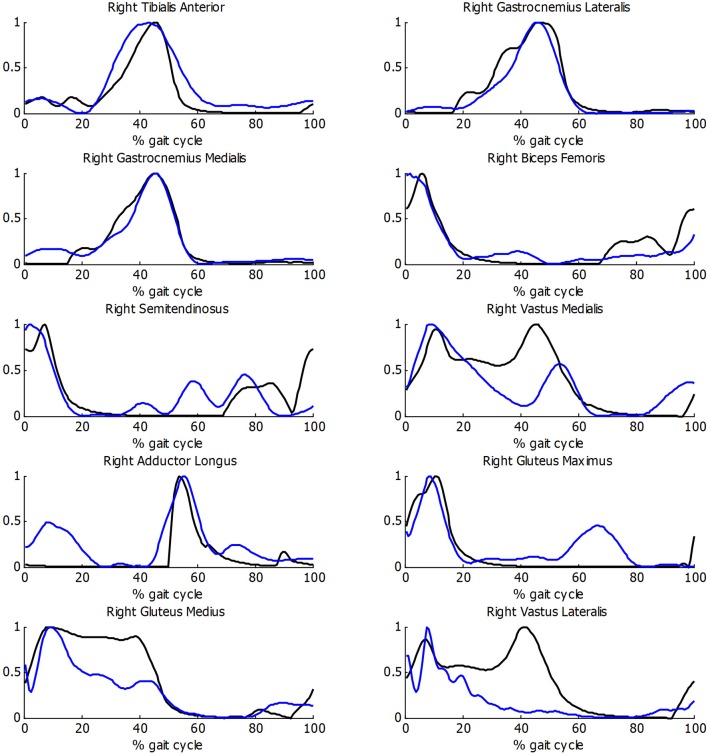
Comparison of normalized muscle activations (black) and normalized EMG measurements (blue) during gait for healthy subject.

Before estimating energy consumption, the musculoskeletal model and the estimation of muscular activity were validated with the EMG measurements for the healthy subject. As there is no clear relationship between EMG amplitude and muscle force (Hof, 1997), the comparison was focused on the shape of the activity patterns, using normalized values. Good correlations between muscular activations and EMG measurements were obtained (Figure 5), with a mean *R* correlation over 0.70.

**Figure 6:**
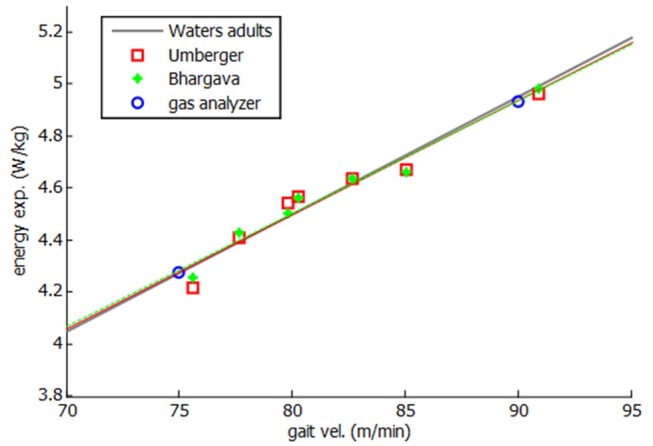
Energy expenditure for healthy subject.

Then, both energy expenditure models were applied and experimentally validated for the healthy subject. Twenty one complete gait cycles were recorded at seven different speeds, ranging between his free selected speed (75 m/min) and his fast speed (90 m/min). As some variability was observed in the obtained values of energy cost for different tests at the same speed, a mean value is represented in Figure 6. Two experimental tests were done, at free selected speed and fast speed, respectively, to validate the models.

As it can be seen in Figure 6, a linear relation was obtained between gait speed and energy consumption, showing a good correlation with both experimental measurements and literature (Waters and Mulroy, 1999). Since a constant discrepancy of the results was observed with respect to the measured energy values, the model was calibrated with such a constant (0.12 W.kg^−1^ for Umberger's model and 1.9 W.kg^−1^ for Bhargava's model). This calibration can be considered as an adjustment of the whole-body basal metabolic rate *k*_*B*_.

On the other hand, three gait cycles were compared for the SCI subject, one with each assistive gait device presented before: (i) passive orthosis owned by the subject; (ii) active orthosis with motor locking the knee; (iii) active orthosis with motor moving the knee. After some few training sessions, the SCI subject was able to walk with confidence wearing the active KAFO, achieving the same self-selected speed of 33 m/min in the three cases.

While the walking speed was the same in the three cases, some kinematic differences could be observed (Table 1). First, the step length, of 45 and 66 cm for the right and left legs, respectively, using the original KAFO, changed to 58 cm for both sides when using the active KAFO with motor moving the knee. The initial circumduction of the left foot (KAFO's leg) of 11.5 cm with the original KAFO was reduced to 7.25 cm thanks to the actuated knee flexion. Pelvic maximum rotations were reduced from −27.6 and 44.8 to −22.5 and 35.3° in the transverse plane, and from 19.18 to 15.23° in the frontal plane. Finally, the mediolateral center of mass (COM) displacement was significantly reduced from 13.48 to 11.54 cm, while the vertical displacement was almost the same in the three cases.

**Table 1:** Comparison of obtained results with the three gait-assistive devices.

		**Passive KAFO**	**Active KAFO** **(locked knee)**	**Active KAFO** **(moving knee)**
Gait velocity (m/min)		33	33	33
Vertical COM displacement (cm)		3.47	3.79	4.11
Mediolateral COM displacement (cm)		13.48	13.42	11.54
Step length (cm)	Right	0.45	0.52	0.58
	Left	0.66	0.62	0.58
Left circumduction (cm)		11.52	9.10	7.25
Range of pelvic rotations in frontal plane (°)		[−4.81; 19.18]	[−4.56; 16.93]	[−4.86; 15.23]
Range of pelvic rotations in transverse plane (°)		[−28.74; 42.87]	[−28.32; 37.93]	[−24.36; 31.83]
Maximum joint reaction forces at shoulders (BW)	Right	1.92	2.13	2.22
	Left	1.91	2.15	2.24
Energy cost (W/kg)	Umberger	3.49	3.56	3.28
	Bhargava	3.11	3.13	3.02

Instrumented crutch measurements did not show significant differences between devices. A mean load of 20% of the bodyweight was observed during the gait cycle, with peaks of 55% (left crutch), and 40% (right crutch) at swing start. Estimated joint reaction forces at shoulder were similar too, with peaks between 190 and 225% (left shoulder) of the bodyweight.

**Figure 7:**
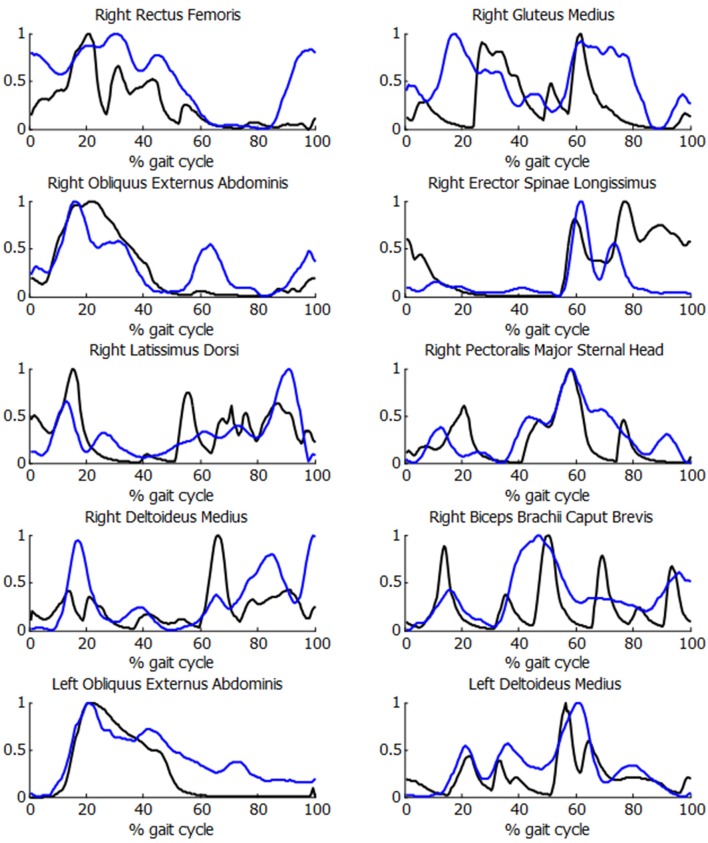
Comparison of normalized muscle activations (black) and normalized EMG measurements (blue) during the crutch-orthosis-assisted gait of a SCI subject.

In order to check the validity of the inputs provided to the energetic cost calculations for the SCI subject, the muscle activations were compared with experimental EMG measurements. As it can be observed in Figure 7, acceptable correlations were obtained, with a mean *R* correlation of more than 0.55.

**Figure 8:**
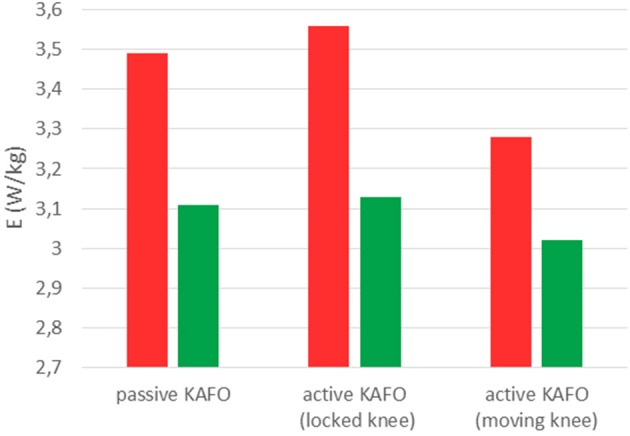
Energy consumptions obtained with Umberger's model (red) and Bhargava's model (green) for the SCI subject wearing the three gait-assistive devices.

Figure 8 and Table 1 show the estimated energy consumptions yielded by both models. The energy cost obtained with the original KAFO was 3.49 W.kg^−1^ for Umberger's model, and 3.11 W.kg^−1^ for Bhargava's. Wearing the active KAFO with motor locking the knee, it was 3.56 and 3.13 W.kg^−1^. Finally, wearing the active KAFO with motor moving the knee, the energy cost was 3.28 and 3.02 W.kg^−1^.

## Discussion

The energy expenditure of a healthy male during gait was calculated, based on the muscular magnitudes obtained from a motion-force-EMG capture and a musculoskeletal model of the subject, through the application of two methods found in the literature (Umberger's and Bhargava's), and was validated by experimental measurements and references from literature for several gait velocities. Results showed that calibration of the methods is necessary to evaluate the whole-body basal metabolic rate. However, the slopes (energy cost vs. gait speed) obtained with both methods were coincident and agreed with those from experiments and literature, which is the essential point to compare two activities performed by the same subject, and using the same model. Based on these findings, both methods were applied to a SCI subject walking with the help of crutches and wearing different gait-assistive devices.

The self-selected gait velocity achieved by the SCI subject with the three devices was of 33 m/min, which is higher than the velocity corresponding to his LEMS (20.2 m/min) according to Waters and Mulroy (1999). This discrepancy can be explained by the moderately strong linear relationship (*R* = 0.64) between walking speed and the LEMS, and by the fact that the subject was tall and athletic.

The SCI subject carried out few training sessions with the active KAFO, and probably needed more experience to show a significant evolution with respect to the passive device, as observed in Font-Llagunes et al. (2016). However, some improvements of the gait pattern thanks to the knee actuation provided by the KAFO were already detected, as symmetry in the step lengths, reduced circumduction and reduced pelvic rotation. COM displacements are generally used as indicators of balance control to reflect the whole body motion during gait. While the vertical displacement was almost the same for the three cases and was close to that of healthy subjects [3.61 cm at 1 m/s (Orendurff et al., 2004)], the mediolateral displacement reflected differences in gait pattern and with respect to healthy subjects [5.96 cm at 1 m/s (Orendurff et al., 2004)].

Ground force reactions measured by the instrumented crutches did not highlight any differences between the devices used, likely because of the short training period with the new device. However, the obtained values showed the demanding use of the upper extremities, which are primarily not designed to walk and to put up with such loads.

Same observations can be done regarding the joint reaction force at shoulders, with estimated peaks >220% of the bodyweight. Westerhoff et al. (2012) reported maximum loads of up to 170% during *in vivo* measurement of shoulder loads during crutch-assisted walking, but subjects were not suffering from any lower limb disability. Highest peaks at the left arm were observed during the left-leg swing (leg wearing the KAFO), likely because the subject needed to compensate the instability of the left foot and the lack of force in the right leg, and to avoid the foot contact with the ground.

Correlations observed between EMG measurements and muscle activations for the SCI subject were acceptable and allow trusting in the input used to calculate the energy cost.

The estimated energy consumptions presented for the SCI subject were not calibrated because the 5-min tests carried out by the healthy subject were not possible for him. Bhargava's results were lower than Umberger's. However, the same order was maintained among the three devices. The active KAFO with locked knee showed the highest value, a bit more than the passive KAFO. This difference could be explained by the additional mass of the motor. The motor actuation reduced significantly (almost 8% for Umberger and 3.5% for Bhargava) the estimated energy consumption despite the short period of training with the device.

As a reference, at the speed developed by the SCI subject (33 m/min), a healthy subject should consume 2.385 W.kg^−1^ (Waters and Mulroy, 1999). Continuing with Waters' references for SCI subjects, for a LEMS of 13, the subject should consume 149.8% more than a healthy subject at the same speed. This would correspond to an energy consumption of 3.57 W.kg^−1^, which is close to the values obtained with Umberger for the two first cases (3.49 and 3.56 W.kg^−1^). In the third case the motor actuation produces the knee flexion/extension, so that the LEMS could be increased to 14. Then the corresponding energy consumption increase should be of 145.5% with respect to a healthy subject, thus leading to a consumption of 3.47 vs. 3.28 W.kg^−1^ obtained with Umberger. While results obtained without calibration are closer to the mentioned references for Umberger's model, slopes (energy cost vs. LEMS) are closer (gradient of −0.1) using Bhargava's model (gradient of −0.11) than Umberger's (gradient of −0.28).

## Conclusion

A method to estimate the energetic cost of the gait of SCI subjects walking with the help of knee-ankle-foot orthosis and crutches has been proposed in this paper. The method just requires to make some motion-force-EMG captures of a subject's gait cycle and, using the generated data, perform an inverse-dynamics analysis, and muscle force sharing optimization on a musculoskeletal model of the subject, so that Umberger's or Bhargava's method can be applied to the obtained results in order to get an estimation of the energy consumption. Therefore, unlike experimental methods reported in the literature which require tests lasting several minutes, the method proposed here only needs that the subject walks during two or three gait cycles, so that one full gait cycle is captured in the gait analysis lab. This makes the method feasible even for the training period, and even for subjects who will not be capable of walking for several minutes after the training period has been completed. However, the advantage may also be a disadvantage, as lower accuracy in the estimation can be expected due to the short duration of exercise on which it is based.

Some limitations can be pointed out in this work. The first limitation is that one single SCI subject was considered in the study, but finding hip-flexion able SCI candidates for actively assisted gait is not easy and developing customized devices for them is expensive and time-consuming. A second limitation is that the SCI subject performed few training sessions with the active orthotic device; it would had been desirable to continue the study for a longer period and see the evolution of the energetic cost as the user became more acquainted with the device.

Future works could go in the direction of overcoming the limitations previously described. Repeating the study for more SCI subjects and spanning longer periods, from the initial training in the use of active orthoses to the stage when a strong skill is attained by the user, would allow to further confirm the validity of the method and its ability to provide a clue, already during the training period, on whether the particular orthotic device will be successful for the particular patient in the mid and long terms.

## Data Availability

The datasets generated for this study are available on request to the corresponding author.

## Ethics Statement

This study was carried out in accordance with the recommendations of guidelines provided by the Committee of Ethics of the University of A Coruña with written informed consent from all subjects. All subjects gave written informed consent in accordance with the Declaration of Helsinki. The protocol was approved by the Committee of Ethics of the University of A Coruña.

## Author Contributions

FlM designed and performed the experiments with the supervision of UL, derived the models, and analyzed the data. FlM and JC wrote the manuscript in consultation with UL and FrM.

### Conflict of Interest Statement

The authors declare that the research was conducted in the absence of any commercial or financial relationships that could be construed as a potential conflict of interest.
